# Roswell Park Medical Club

**Published:** 1902-01

**Authors:** George F. Cott

**Affiliations:** Secretary


### Roswell Park Medical Club.

REPORTED BY GEORGE F. COTT, M. D., Secretary.

*Monday, November 4, 1901.*

PRESENTATION OF CASES.—SARCOMA OF KIDNEY.

Dr. BAGLEY.—A little girl two years old, very sick, some temperature, distended abdomen, rapid pulse, abdominal veins prominent, everything indicative of tubercular condition. Dr. Kenerson resected two ribs but could not satisfy himself as to condition within. Suspected tumor of the pleura. Child was taken home; did not improve and laparatomy was performed. Found large sarcoma of left kidney. Death occurred soon afterward.

NEW SYMPTOM IN APPENDICITIS.

Dr. GIBSON.—A peculiar symptom occurring in appendicitis. Patient young woman between 20 and 30. Most prominent and persistent symptom and which among others was pain in the region of the knee. She was treated by two doctors for diseases of the knee; did not improve. A surgeon manipulated the abdomen and found a tumor in location of the appendix. Appropriate treatment afforded complete relief. He ascribed the localisation of the pain to pressure on the filaments of the anterior crural nerve, which arises from the 2d, 3d and 4th lumbar segments and which furnish filaments to anterior femoral region or branch to knee joint.

CATHETER PENETRATING WALL OF UTERUS.

Dr. KRAUSE.—Woman aborted herself at second month with catheter. Bled nearly three months; miscarried at the fifth.

Everything went on well until seven weeks after miscarriage, when I was called on account of vomiting. Had been troubled since miscarriage by constipation; now greatly relieved. Vomiting nearly ceased in about twenty-four hours; in another twenty-four became more persistent, tympanites appeared; no movement of bowels. Diagnosis of obstruction. Operation revealed an adhesion between omentum and uterus where catheter had penetrated the wall. Patient confessed abortion. Recovered.

Dr. Wende.—Reported the case of a young woman who aborted herself with soft catheter and lost it. Thought she passed it through the uterus and began to vomit. Operation per vaginam, but nothing found. Girl died. No post mortem.

